# Differences in fall-related characteristics across cognitive disorders

**DOI:** 10.3389/fnagi.2023.1171306

**Published:** 2023-06-09

**Authors:** Karolina Minta, Giorgio Colombo, William R. Taylor, Victor R. Schinazi

**Affiliations:** ^1^Future Health Technologies, Singapore-ETH Centre, Campus for Research Excellence and Technological Enterprise (CREATE), Singapore, Singapore; ^2^Department of Pharmacology, Yong Loo Lin School of Medicine, National University of Singapore, Singapore, Singapore; ^3^Department of Health Sciences and Technology, Institute for Biomechanics, ETH Zürich, Zürich, Switzerland; ^4^Department of Psychology, Bond University, Gold Coast, QLD, Australia

**Keywords:** cognitive impairment, dementia, injurious falls, fall prevention strategies, fall risk, gait

## Abstract

Approximately 40–60% of falls in the elderly lead to injuries, resulting in disability and loss of independence. Despite the higher prevalence of falls and morbidity rates in cognitively impaired individuals, most fall risk assessments fail to account for mental status. In addition, successful fall prevention programmes in cognitively normal adults have generally failed in patients with cognitive impairment. Identifying the role of pathological aging on fall characteristics can improve the sensitivity and specificity of fall prevention approaches. This literature review provides a thorough investigation into fall prevalence and fall risk factors, the accuracy of fall risk assessments, and the efficacy of fall prevention strategies in individuals with diverse cognitive profiles. We show that fall-related characteristics differ between cognitive disorders and fall risk assessment tools as well as fall prevention strategies should critically consider each patient’s cognitive status to facilitate the identification of fallers at an earlier stage and support clinical decision-making.

## 1. Introduction

The Prevention of Falls Network Europe (ProFANE) group defines a fall as “an unexpected event in which a person comes to rest on the ground, floor, or lower level” (Lamb et al., 2005). Falls are one of the most common healthcare problems among the elderly. It is estimated that one-third of elderly individuals above 65 years of age fall at least once per year (Talbot et al., 2005). Fall incidents steadily increase with age, and affect up to 50% of adults over the age of 80 (Inouye et al., 2009). While 30% to 50% of falls only result in minor lesions such as bruises or lacerations, between 5 and 10% lead to more severe injuries such as fractures or traumatic brain injury (Masud and Morris, 2001). Another challenge is the progressive development of fear of falling, that often restricts activities of daily living, leading to social isolation and depression. Falls also pose a serious economic burden for healthcare systems (Haddad et al., 2019). Every year, approximately USD 50 billion are spent on medical expenses attributed to fall-related injuries (Florence et al., 2018), highlighting the economic need for healthcare systems to focus on effective fall prevention strategies. To tackle this challenge, it is critical to identify characteristics related to fall risk that can help healthcare practitioners prescribe effective intervention programs to reduce and prevent injurious falls.

Falls are closely related to cognitive dysfunction (Holtzer et al., 2007). Indeed, individuals with cognitive impairment exhibit a higher risk of falls. For example, it is estimated that patients with dementia experience up to eight times more falls but also have a lower rate of recovery compared with patients without dementia (Allan et al., 2009). Although a large body of research has established predictors of falls and their consequences in healthy aging (Rawsky, 1998; Borycki, 2000; Karlsson et al., 2013; Hacidursunoglu Erbas et al., 2021), it remains unclear whether fall-related characteristics differ according to the level of cognitive disability (e.g., Mild Cognitive Impairment (MCI), Alzheimer’s Disease (AD), Cerebrovascular Disease (CVD), Vascular Dementia (VaD), Dementia with Lewy Bodies (DLB), Parkinson’s Disease (PD), Normal Pressure Hydrocephalus (NPH), Huntington’s Disease (HD), etc.). Since fall prevention strategies that are successful in cognitively normal adults have generally failed to reduce fall risk in people with cognitive impairment (Shaw, 2007), identifying the role of pathological aging on fall characteristics could improve the efficacy of fall prevention approaches. To our knowledge, no review has yet been undertaken that highlights the contribution of different cognitive disorders to fall risk factors and the associated efficacy of prevention strategies. The purpose of this review is, therefore, to identify the differences in fall prevalence and fall risk factors, as well as the accuracy of fall risk assessments and efficacy of fall prevention strategies, across patients with different cognitive profiles.

## 2. Fall characteristics

### 2.1. Fall risk factors

There are over 400 recognized risk factors for falling, although no reliable consensus or classification system has been agreed (Masud and Morris, 2001). In this review, we distinguish fall risk factors identified through observational studies, clinical trials and statistical analyses from the unique symptoms of each disorder (e.g., freezing of gait in PD patients). The majority of falls are associated with multiple risk factors, and the likelihood of falling is thought to increase with an increasing number of comorbidities (Tinetti et al., 1988). Impaired gait and balance are widely known to be important risk factors for falls (Hausdorff et al., 2001; Brach et al., 2005; Verghese et al., 2009). Changes in gait patterns are very common in the older population, with approximately 35% of adults aged 70 and over being diagnosed with abnormal gait (e.g., neuropathic, parkinsonian, or spastic gait) (Verghese et al., 2006). This prevalence rapidly increases with age, where walking patterns in older adults tend to be slower, stiffer and less coordinated, with a shorter stride length and lower toe-clearance (Jensen et al., 2001). While these reported key fall characteristics are strongly associated with physical movement patterns, motion coordination and balance are exclusively driven by neurological signaling. Importantly, however, the role of cognitive status on fall risk is often overlooked, even though the rate of falls is eight times higher in individuals with cognitive impairment than in their cognitively intact counterparts (Allan et al., 2009). This argument is supported by the continued high prevalence of falls among dementia patients despite relatively intact motor function, highlighting that falls do not always directly result from impaired gait and balance (van Iersel et al., 2006).

Impairments in executive function (e.g., self-control) and dual-tasking (e.g., walking and talking) are commonly associated with falls (Hsu et al., 2012; Mirelman et al., 2012). Indeed, fallers perform significantly worse in tests of executive function compared to non-fallers (Springer et al., 2006; Holtzer et al., 2007). Specifically, individuals who score lower on tests of executive function at baseline are three times more likely to fall in the next 2 years, but also transition faster to become fallers (Herman et al., 2010). In addition, worse scores for executive attention are associated with single and recurrent falls (Holtzer et al., 2007). Furthermore, impairment in executive function reduces an individual’s attention to balance and gait, and ability to adapt to environmental obstacles, and may lead to an increased fall risk. Overall, lower executive function has been associated with mobility decline after a fall (Hughes et al., 2020). In contrast to executive function, the relationship between other measures of cognitive function (e.g., memory) and falls remains controversial. For example, Huang et al. (2022) reported that memory deficits (especially poor short-delayed memory) assessed by the auditory verbal learning test are associated with increased fall risk, and this association might be mediated by the atrophy of medial temporal, frontal, and parietal lobes. However, other studies have not observed this relationship (Hausdorff et al., 2006; Springer et al., 2006; Holtzer et al., 2007). The differential relation of distinct measures of cognitive function (executive function and memory) to falls may depend on the neural structures that are recruited. The unique associations between executive function and falls suggest that the frontal basal ganglia circuitry, which mediates executive control processes (D’Esposito et al., 1995) and plays a critical role in motor control (Yamada et al., 2016), may have an impact on fall risk. These findings suggest that fallers may have specific cognitive processing deficits rather than a global decline in cognitive function. Here, executive function deficits are defining features of the cognitive profile in fallers and are, therefore, potential targets for fall risk screening and interventions.

In dual-tasking conditions, the inability to maintain a conversation (Lundin-Olsson et al., 1997) or a variation in verbal task performance (Verghese et al., 2002; Bootsma-van der Wiel et al., 2003; Beauchet et al., 2007), while walking have been reported as markers for future falls, suggesting an involvement of attention in gait control. Interestingly, the occurrence of falls has been associated with both poorer and improved spoken task performance. For example, slower reciting of the alphabet (Verghese et al., 2002) and lower number of recited words (Bootsma-van der Wiel et al., 2003) but faster counting backward (Beauchet et al., 2007) and a higher number of enumerated figures (Beauchet et al., 2007) while walking are all considered to be predictors of falls. Similarly, dual-tasking may have both positive and negative effects on gait itself. For instance, simple cognitive tasks (e.g., 1-back) when walking might improve the rhythmicity of gait patterns (Verrel et al., 2009), but more complex dual-tasking results in slower walking, fewer steps, and increased stride time variability, leading to increased fall risk (Hunter et al., 2020). Overall, the association of falls with poorer spoken task performance may result from the interference caused by competing demands for attentional resources between gait and verbal tasks (Woollacott and Shumway-Cook, 2002). A possible explanation for the association of falls with the improved spoken task performance, such as a positive counting performance score, is that similar to walking, counting backward is a task that includes a strong rhythmic component. Here, some authors suggest that the combination of two simultaneously performed rhythmic tasks could synergistically influence each other (Beauchet et al., 2007).

Extensive reviews of fall risk factors have been presented previously (Tinetti et al., 1988; Landi et al., 2005; Talbot et al., 2005; Dionyssiotis, 2012; Salonen and Kivela, 2012; Quach et al., 2019; Kim et al., 2020). In addition to increasing age, impaired gait, and cognitive dysfunction, other factors, including gender (Talbot et al., 2005), history of falls (Tinetti et al., 1988), visual status (Salonen and Kivela, 2012), social engagement (Quach et al., 2019), socioeconomic status (Kim et al., 2020), nutritional deficiencies (Dionyssiotis, 2012), and use of psychotropic medication (Landi et al., 2005) have all been identified as risk factors for falling. In this review, we aim to comprehensively understand the relationships between cognitive status and fall risk and will therefore not go further into details on other physiological and psychological risk factors.

### 2.2. Fall risk assessments

Fall risk assessments are designed to identify individuals at increased risk of falling, determine the associated risk factors, and indicate the most efficient fall prevention strategies. To date, some 20 scales already exist for identifying fall risk (Perell et al., 2001), resulting in a lack of consensus on the most appropriate scale. The most widely used measure of fall risk in both clinical and research settings is the Timed Up and Go (TUG) test (Podsiadlo and Richardson, 1991). Other commonly used fall risk assessment tools focus either only on balance deficits [BBS: Berg Balance Scale (Berg et al., 1992), FAB: Fullerton Advanced Balance (Rose et al., 2006)], balance and gait [TMT: Tinetti Mobility Test (Tinetti, 1986)] or multiple domains of physical function [PPT: Physical Performance Test (Wilkins et al., 2010)]. However, the ability of these tests to predict falls in elderly populations is limited, with a sensitivity ranging between 31 and 79% and specificity between 52 and 74% (VanSwearingen et al., 1998; Raiche et al., 2000; Hernandez and Rose, 2008; Barry et al., 2014; Park and Lee, 2017). Given that most falls risk assessment tools have relatively low predictive performance, other ways of assessing fall risk should be considered. Neuropsychological evaluation may be a potential candidate as it is efficient, inexpensive, and flexible. Surprisingly, the most common fall risk assessments have failed to account for mental status, despite the demonstrated influence of cognitive processes on fall risk (Holtzer et al., 2007). Previous research has shown that poor performance on cognitive tests is associated with increased likelihood of falling among older adults (Holtzer et al., 2007; Gleason et al., 2009). For example, people who score lower on the Mini-Mental State Examination (MMSE), a widely used screening tool for cognitive impairment, are most prone to fall (Gleason et al., 2009). However, to the best of our knowledge, no study has been conducted to validate the accuracy of such neuropsychological assessments in fall prediction.

### 2.3. Fall prevention strategies

Previous research has identified several fall prevention strategies among elderly people that include cognitive (Verghese et al., 2010; van het Reve and de Bruin, 2014; Smith-Ray et al., 2015) and physical (Wolf et al., 1996; Li et al., 2005; Sherrington et al., 2008; Barker et al., 2016) training. While cognitive measures are not specifically included in most common fall risk assessments, cognitive training is thought to be essential for effective fall prevention as cognitive processing plays a crucial role in gait and balance (Verghese et al., 2010), and thereby has the potential to reduce falls (Verghese et al., 2010; van het Reve and de Bruin, 2014; Smith-Ray et al., 2015). In particular, game-based cognitive training has received increasing attention in supporting healthy aging and fall prevention. For instance, participants that completed a computer-based cognitive training that targets various cognitive domains, exhibited improved performance in the TUG test (Smith-Ray et al., 2015). Another computerized cognitive remediation consisting of multiple visual, auditory, and cross-modality tasks has shown a 22% improvement on the dual-walking-while-talking task (Verghese et al., 2010).

A meta-analysis of 44 randomized controlled trials showed that physical training addressing strength, balance, endurance, flexibility and walking can reduce fall rates in the elderly population by approximately 17% (Sherrington et al., 2008). In total, their analysis involved nearly 10,000 participants suggesting studies with robust findings that are generalizable to a broad proportion of older people. However, it is important to note that this meta-analysis included studies on different types and intensities of exercises, which can lead to discordant conclusions. More recently, a systematic review of 116 studies showed a variation in the effects of different types of exercise on fall rates. For example, balance and functional exercises were shown to reduce the number of falls by 24%, while programs combining balance, functional and resistance exercises by 28% (Sherrington et al., 2020). Importantly, however, training programs that combine body movement and mental status have been found to be significantly more effective. Specifically, some of the randomized controlled trials showed that Tai Chi and Pilates can reduce the risk of falls by up to 55% (Wolf et al., 1996; Li et al., 2005) and 64% (Barker et al., 2016), respectively. Furthermore, virtual-reality balance exercises in a clinical randomized controlled trial performed on 60 elderly individuals have been associated with improved performance in the TUG by 9% and BBS by 15% tests, as well as a diminished fear of falling by 11% (Zahedian-Nasab et al., 2021). Dance-based interventions that improve motor and cognitive skills, such as Korean traditional dance (Jeon et al., 2005), Thai traditional dance (Noopud et al., 2019), jazz (Alpert et al., 2009), and Turkish folkloric dance (Eyigor et al., 2009), have all been recommended to be utilized in falls prevention programs. As the majority of dance-based interventions are only pilot studies, their outcomes should be used as a foundation to conduct larger studies targeting more varied populations. Although this body of research strongly suggests that adding a cognitive component to physical exercise may enhance the efficacy of fall prevention strategies, no previous studies have validated the effectiveness of cognitive training for reducing the number of falls, and therefore the level of evidence for recommending preventive interventions based on cognitive training remains low.

## 3. Falls across cognitive disorders

It is widely acknowledged that the risk of falling is associated with cognitive dysfunction (Holtzer et al., 2007). However, to our knowledge, no review has yet been undertaken that highlights the contribution of different cognitive disorders to fall prevalence, fall risk factors, fall risk assessments, and fall prevention strategies. To address this deficit, we aimed to conduct a comprehensive review of the literature in order to understand the role of cognitive status on fall risk. To achieve this, we searched the PubMed database using the term “fall risk” together with the name of each cognitive disorder (“mild cognitive impairment,” “Alzheimer*,” “cerebrovascular disease,” “vascular dementia,” “dementia with Lewy bodies,” “Huntington*,” “normal pressure hydrocephalus,” “Parkinson*”) either present in the title or abstract fields. The purpose of this review was to identify the differences in fall-related characteristics (i.e., fall prevalence and fall risk factors, as well as the accuracy of fall risk assessments and efficacy of fall prevention strategies) across patients with different cognitive profiles with the aim of improving the sensitivity and specificity when prescribing fall-prevention strategies. Other cognitive disorders, such as frontotemporal dementia, progressive supranuclear palsy, multiple system atrophy, corticobasal degeneration, and Creutzfeldt-Jakob disease, all present clear gait, balance, and cognitive deficits, but their low prevalence and lack of evidence-based scientific research into fall risk assessment and prevention strategies place these diseases beyond the scope of this review.

### 3.1. Mild cognitive impairment

Mild cognitive impairment is clinically defined as a deficit in at least one cognitive domain (e.g., episodic memory) in the absence of dementia. MCI is considered to be a preclinical stage of dementia with an annual conversion rate of 10% (Mitchell and Shiri-Feshki, 2009). Patients with MCI can be categorized as amnestic MCI (a-MCI) and non-amnestic MCI (na-MCI). While a-MCI is characterized by memory deficits and is associated with a higher rate of conversion to AD, na-MCI patients have impairments in other domains, such as language, attention, or spatial ability, and have a higher risk to convert to other types of dementia (e.g., DLB) (Csukly et al., 2016).

Although people with MCI maintain relatively good physical function and have only subtle cognitive impairment, previous research has shown that they fall nearly twice as often as healthy individuals (Borges Sde et al., 2015; Goncalves et al., 2018; Ansai et al., 2019), which is presumed to be the result of neurocognitive changes, including decline in executive function. Specifically, MCI patients also show poor dual-task performance (Ansai et al., 2019), which is considered to be the most sensitive tool for early identification of MCI-related motor deficits (Bahureksa et al., 2017). For example, the TUG test administered along with a secondary motor-cognitive task revealed that MCI fallers take longer and require more steps than non-fallers (Goncalves et al., 2018).

People with MCI often exhibit symptoms of slowness in gait, decreased stride length, increased stride time variability, and poor balance (Makizako et al., 2013; Montero-Odasso et al., 2014). Furthermore, a-MCI patients have more severe deficits in gait velocity and stride time variability compared with the na-MCI subtype (Montero-Odasso et al., 2014), even though fall risk is thought to be greater in people with na-MCI (Delbaere et al., 2012). This finding suggests that factors other than gait abnormalities contribute to the increased risk of falling and are more specific to na-MCI. For instance, it cannot be excluded that impairments in cognitive domains such as language, attention, or spatial ability commonly present in na-MCI (Csukly et al., 2016) are possible risk factors for falling in this subgroup. MCI patients do exhibit greater fear of falling than cognitively healthy individuals (Uemura et al., 2014). Anxiety, low self-esteem, and depression are common behavioral symptoms in MCI and significant contributors to increased prevalence of fear of falling (Kalbe et al., 2005; Van der Mussele et al., 2013; Uemura et al., 2014; Ansai et al., 2019).

Pathological mechanisms relating MCI and falls are largely unexplored. Snir et al. (2019) reported that poor white matter integrity is associated with increased risk and history of falls in MCI. Interestingly, the integrity of white matter microstructure in the tracts involved in executive and visuospatial functions are mainly affected in MCI patients with a history of falls. Furthermore, multiple studies have found that reduced volume of various brain regions (e.g., hippocampus) are associated with high fall risk in MCI patients (Allali et al., 2016, 2020; Huang et al., 2022).

Several fall prevention strategies have demonstrated success in reducing risk factors and rate of falls in MCI patients. For instance, Tai Chi training reduced the PPA measures by up to 88% (Sungkarat et al., 2017). Similarly, a multicomponent exercise program involving aerobic, resistance, and balance exercise was found to improve attention, dual-task ability, and reduce risk of falling by 60% (Thaiyanto et al., 2021). Fischer (2021) reported that fall prevention programs solely based on cognitive tasks reduce fall rates, with a median decrease of two falls per person over a 6-month period. In addition, sensor-based interactive balance training reduced fear of falling in MCI patients by 9% and may lead to an increased self-efficacy in avoiding falls (Schwenk et al., 2016). Finally, Montero-Odasso et al. (2019) presented a concept for reducing falls in MCI patients via donepezil treatment, which also improved dual-task gait speed.

### 3.2. Alzheimer’s disease

Alzheimer’s disease is a progressive neurodegenerative disorder and the most common form of dementia, affecting millions of individuals worldwide, and is characterized by a decline in memory, orientation, and attention, among other symptoms. Individuals with AD are two to three times more likely to fall compared with those who have normal cognition (Imamura et al., 2000; Allan et al., 2009), and it is estimated that 50% of patients with AD will fall in their lives (Ansai et al., 2019). However, the fall prevalence between mild and moderate stages of AD remains controversial (Kato-Narita and Radanovic, 2009; Coelho et al., 2012), suggesting that further research is required in this area.

In addition to impaired cognitive function, other symptoms in AD include gait alterations and deterioration in motor ability, placing these patients at even greater risk for falls (Ryan et al., 2011). Interestingly, although falls in AD patients have been associated with neuroleptic drug use and visuospatial deficits (e.g., numerosity perception or visual closure) (Horikawa et al., 2005; Ansai et al., 2019; Oki et al., 2021), this relationship was not found in MCI patients (Ansai et al., 2019). Deficits in visuospatial abilities in AD patients may thus be associated with difficulties in avoiding path obstacles and consequently result in a higher risk of falling. Critically, dual-task tests, although a promising predictor of falls in MCI patients, are not a predictor of falls in AD patients since no differences in dual-task performance have been found between fallers and non-fallers (Goncalves et al., 2018; Ansai et al., 2019). Furthermore, AD patients show a much lower prevalence of fear of falling compared with MCI (Borges Sde et al., 2015), which is plausibly associated with the differences in anxiety levels between the two groups (Van der Mussele et al., 2013) and the fact that AD patients suffer from anosognosia (Kalbe et al., 2005).

Pathological mechanisms relating AD and falls are largely unexplored. Fallers with AD present a higher grade of periventricular white matter lesions (Horikawa et al., 2005) and smaller hippocampal volumes (Keleman et al., 2020) compared with AD non-fallers. Increased risk of falling may be detected in AD using Positron Emission Tomography (PET) imaging or Cerebrospinal Fluid (CSF) biomarkers (Stark et al., 2013), where higher levels of Pittsburgh compound B retention (reflecting amyloid depositions in the brain) and CSF t-tau (reflecting neuronal damage) are associated with a faster time to first fall (Stark et al., 2013). Since treatment of AD symptoms has a positive effect on attention and executive function, it would be reasonable to assume that such therapeutics could also reduce falls. However, patients on AD medication have approximately 60% greater chance of falling than those not treated (Epstein et al., 2014). Thus, it is important to understand the possible adverse effects of medication on motor coordination.

Fall risk assessments in AD remain relatively understudied. Although the PPT can be used for assessing function and frailty in AD and has shown potential for identifying AD patients with a history of falls, research has shown that this assessment is incapable of predicting falls (Farrell et al., 2011; Ryan et al., 2011). Regarding fall prevention strategies in AD patients, multicomponent training programs involving balance, dance, strengthening and walking exercises (Suttanon et al., 2013), or a combination of motor and cognitive exercises (Hernandez et al., 2010; Abreu and Hartley, 2013) have all been found to improve functional mobility and also reduce falls. Indeed, Pitkala et al. (2013) have found that AD patients who perform physical exercises twice a week over a year experience up to 52% fewer falls than those who lead more sedentary lifestyles.

### 3.3. Cerebrovascular disease and vascular dementia

Cerebrovascular disease is caused by a reduced supply of cerebral blood flow to the brain due to blood vessel damage, eventually leading to VaD. Following AD, VaD is the second most common form of dementia (Dubois and Hebert, 2001), although clinical symptoms differ between the two. In contrast to AD, gait disturbances are commonly apparent in the early stages of VaD (Jamour et al., 2012). Furthermore, AD is characterized by greater impairment in episodic memory, while VaD patients have greater deficits in attention and visuospatial function (Graham et al., 2004).

The prevalence of falls in VaD is similar to AD and occurs in approximately 50% of patients (Allan et al., 2009). The fall rate in CVD, including stroke, is lower than in VaD and ranges between 11 and 37% (Nyberg and Gustafson, 1997; Tutuarima et al., 1997; Sze et al., 2001; Teasell et al., 2002; Chaiwanichsiri et al., 2006). The main risk factors for falls in CVD include postural sway, paralysis, hypoesthesia, visual deficits, impulsivity, heart disease, urinary incontinence, cognitive decline, and hemineglect (Sackley, 1991; Rapport et al., 1993; Tutuarima et al., 1997; Tsur and Segal, 2010). Pathological changes in VaD have also been linked to falling. Here, higher amyloid plaque deposition (Dao et al., 2017) and a greater progression of white matter hyperintensities (Callisaya et al., 2015) in VaD patients were found to be associated with a higher risk of falling and an increased risk of repeated falls, respectively.

Due to the high frequency of recurrent falls in post-stroke patients, several scales have been developed to identify individuals at risk of falls (Breisinger et al., 2014; Guimaraes et al., 2020; Yang et al., 2021). The Stroke Assessment of Fall Risk (SAFR) (Breisinger et al., 2014; Yang et al., 2021), which includes items related to stroke-specific neurologic deficits, has demonstrated the highest sensitivity and specificity (78 and 63%, respectively) when compared with other fall risk assessments, such as the Recurrent Fall Risk (ReFR) scale (Guimaraes et al., 2020), Morse Fall Scale (Yang et al., 2021), and Fall Harm Risk Screen (Breisinger et al., 2014). Efficacy of fall prevention strategies has also been evaluated in CVD. Here, both Zhou et al. (2021) and Spano et al. (2022) developed a motor-cognitive rehabilitation program that was found to improve balance, gait, and reduce fear of falling as well as falls in CVD patients. In addition, virtual reality training has shown promise in improving the TUG and BBS scores in post-stroke patients (Park et al., 2017). Surprisingly, while both fall risk assessments and fall prevention strategies have been extensively studied in CVD, there remains a lack of understanding of these characteristics in VaD, despite its considerably higher fall rate.

### 3.4. Dementia with Lewy bodies

Dementia with Lewy bodies is a degenerative neurological disorder, accounting for approximately 20% of dementia cases (McKeith et al., 1996), and characterized by the presence of intra-neuronal aggregates consisting of the synaptic protein α-synuclein in the form of Lewy bodies (McKeith et al., 2004). DLB symptoms include memory and executive dysfunctions, visuospatial impairment, visual hallucinations, parkinsonism, and psychiatric dysfunction (McKeith et al., 2005; Hamilton et al., 2008). Although DLB and AD patients do not differ in rate of cognitive decline (Williams et al., 2006), the progression and prognosis of DLB are worse compared with AD (Bostrom et al., 2007).

Falls are common place in DLB, occurring in more than 70% of patients (Allan et al., 2009), and repeated falls are a part of consensus criteria for the clinical diagnosis of DLB (McKeith et al., 1996). Compared with healthy controls, DLB patients exhibit approximately 40 times worse gait stability and experience six times more falls (Allan et al., 2009). The incidence of falls in DLB is also three times higher than in AD or VaD (Allan et al., 2009), with the incidence of fall-related injuries being ten times higher than in AD (Imamura et al., 2000). In addition, DLB patients have a higher incidence of multiple falls than AD patients, and it has been suggested that five or more falls within 3 months may be a useful diagnostic indicator to discriminate DLB from AD (Ballard et al., 1999). Here, it is believed that the increased falls risk in DLB patients is related to more severe visuospatial and executive deficits, hallucinations, sleepiness, and orthostatic hypotension compared with AD patients (Scharre et al., 2016). Furthermore, motor features (e.g., pace, stride length, and postural control deficits) that distinguish DLB from AD might explain the differences observed in fall-related characteristics between the two groups (Fritz et al., 2016). While DLB is known to affect several different brain regions, to our knowledge no studies have been performed to evaluate the underlying etiology of neural substrates in relation to falls. Despite this lack of knowledge, the Fall Risk Index-21 (FRI-21) has shown efficacy in screening for DLB and differentiating from AD (Tsujimoto et al., 2021). Surprisingly, despite a high frequency of falls in DLB, no specific prevention strategies have been presented.

### 3.5. Parkinson’s disease

Parkinson’s disease is associated with degeneration of the dopaminergic pathway in the basal ganglia, with key clinical symptoms of bradykinesia, rest tremor, rigidity, memory disturbances, and executive dysfunction. When PD patients experience significant cognitive decline after at least a year of motor symptoms, the condition is recognized as Parkinson’s Disease Dementia (PDD) (Allan et al., 2005).

The prevalence of falls in PD and PDD is exceptionally high, with some 51–68% of PD patients (Gray and Hildebrand, 2000; Bloem et al., 2001; Wood et al., 2002) and up to 90% of PDD patients (Allan et al., 2009) becoming fallers. More than 30% of PD patients experience a serious fall-related injury, such as a fracture or intracranial hemorrhage (Auyeung et al., 2012). Although falls are common in the elderly population, even early PD patients younger than 50 years are already at high risk for falls (Voss et al., 2012). Compared with other types of dementia, individuals with PDD have the highest fall rates. The fall incidence in PDD is 20 times higher than in healthy controls, ten times higher than in AD or VaD, and three times higher than in DLB (Allan et al., 2009). Many PD patients also develop a fear of falling, reaching up to 60%, which has been mainly attributed to walking deficits, balance problems, fatigue, turning hesitations, akinesia, and motor fluctuations (Nilsson et al., 2012). Indeed, increased postural sway and gait disturbances are the leading risk factors for falls in PD, which tend to worsen under dual-task conditions (Matinolli et al., 2009).

Several fall-related factors are specific to PD and different from the general elderly population (Gray and Hildebrand, 2000; Paul et al., 2014; Fasano et al., 2017). For instance, freezing of gait is a distinctly abnormal gait pattern for PD and a common cause of falls in this group (Gray and Hildebrand, 2000; Paul et al., 2014; Fasano et al., 2017). In addition, motor fluctuations (Fasano et al., 2017) and dyskinesia (Paul et al., 2014) are absent in the general population but present in PD. Furthermore, posture is usually normal or mildly stooped in healthy individuals, while trunk flexion in PD increases the risk of forward falls (Fasano et al., 2017). Finally, increased fall risk in PD patients is also related to rigidity or reduced leg muscle strength (Paul et al., 2014), loss of arm swing (Wood et al., 2002), impaired cognition (Paul et al., 2014), and orthostatic hypotension (Gray and Hildebrand, 2000; Scharre et al., 2016). A combination of balance and gait assessments, such as the TMT (Kegelmeyer et al., 2007; Contreras and Grandas, 2012), BBS (Schlenstedt et al., 2016), FAB (Schlenstedt et al., 2016), and Hoehn and Yahr staging (Contreras and Grandas, 2012) tests are commonly used to assess fall risk in this population, with sensitivity and specificity ranging between 64 and 77% and 58–79%, respectively. Falls in PD patients are likely to arise from dysregulation and degeneration of neuronal systems, such as the basal forebrain cholinergic projection or the striatal dopamine systems (Sarter et al., 2014). Furthermore, PD patients with a fall history have decreased gray matter volumes in cerebellar lobules (Morelli, 2022). White matter changes that are associated with an increased risk of falls are also common among patients with PD (Lee et al., 2010). Interestingly, PDD patients have significantly increased white matter hyperintensities, especially in the periventricular region compared to PD patients without dementia (Lee et al., 2010). These findings suggest that an influence of cognition on fall risk might originate from white matter lesions.

Regarding the effects of fall prevention programs in PD patients, conflicting results have been presented in the literature. Although some training programs based on balance and strengthening exercises were found not to reduce falls (Canning et al., 2015; Matchar et al., 2017; Chivers Seymour et al., 2019), other studies have reported higher rates of efficacy (Smania et al., 2010; Gao et al., 2014; Morris et al., 2015; Song et al., 2017; Peter et al., 2020; Silva et al., 2021). Here, effective therapeutic interventions in reducing fall rates involve Argentine tango (Peter et al., 2020), dual-tasking sessions (Silva et al., 2021), rehabilitation programs combining movement and strength training (Morris et al., 2015), Tai Chi (Gao et al., 2014; Song et al., 2017), Qigong (Song et al., 2017), and balance training (Smania et al., 2010). Numerous pharmacological options are also available for reducing PD-specific fall risk factors (e.g., medical treatment using levodopa can decrease the duration of gait freezing episodes and their frequency) (Giladi, 2008). Although the incidence of PD has clearly driven effective assessment and prevention programs, further investigation into mitigating the extremely high fall rates associated with PDD is critically required.

### 3.6. Normal pressure hydrocephalus

Normal pressure hydrocephalus is characterized by enlarged ventricles, normal intraventricular pressure, and a typical triad of symptoms: gait disturbances, cognitive decline and impaired bladder control (Hakim and Adams, 1965). NPH is one of the few causes of dementia that can be reversed with treatment through ventricular shunting (Hakim and Adams, 1965). There are two forms of NPH: idiopathic NPH (iNPH; no identifiable cause) and symptomatic NPH (sNPH; resulting from brain infection, hemorrhage, stroke, or brain injury).

Patients with NPH are at increased risk of falling, plausibly related to motor and cognitive impairments (Davis et al., 2021). Compared with healthy individuals, iNPH patients are 15 times more likely to fall, and experience higher fear of falling and lower confidence in avoiding falls (Larsson et al., 2021). Falls in iNPH patients are associated with greater temporal gait variability and worse dynamic, but not static balance function (Nikaido et al., 2019). Although muscle weakness is a well-known fall risk factor, no differences exist in lower limb muscle strength between faller and non-faller iNPH patients (Nikaido et al., 2019). Cognitive impairment may also be a factor influencing the fall rate among iNPH patients, as recurrent fallers present worse cognition and more symptoms of depression (Larsson et al., 2021). Pathological changes in iNPH have also been linked to falling. Here, iNPH patients with small pre-operative anterior callosal angle are at high fall risk. However, after ventriculo-peritoneal shunting, more than 70% of iNPH patients show an improvement in gait and balance as well as fall risk (Mantovani et al., 2021), albeit not recovering to the levels of controls (Larsson et al., 2021).

Although the TUG assessment appears not to be sensitive for fall risk prognosis in NPH patients, the Fall Risk Questionnaire (FRQ) has shown efficacy in predicting fallers, with 96% sensitivity and 48% specificity (Davis et al., 2021). Despite considerable investigation into fall risk in iNPH, almost no work has focused on sNPH and further studies are required in this area.

### 3.7. Huntington’s disease

Huntington’s disease is an autosomal-dominant, progressive, neurodegenerative disorder, where patients present chorea, behavioral changes, psychiatric disorders, and cognitive decline that can progress toward dementia (Ross et al., 2014). Gait impairments are characterized by bradykinesia, hypo/hyperkinesia, reduced velocity, increased variability in spatiotemporal features, and balance impairments (Vuong et al., 2018). Up to 90% of HD patients fall at least once every 6 months (Kegelmeyer et al., 2021), where recurrent fallers are common (up to 60%) (Grimbergen et al., 2008; Busse et al., 2009), but research has surprisingly shown that only 15% of patients are afraid of falling (Grimbergen et al., 2008), plausibly explainable by generally reduced fear ratings in response to fear stimuli (Eddy et al., 2011) and general lack of awareness (McCusker and Loy, 2014). Compared with non-fallers, HD-fallers have decreased gait velocity and stride length, increased trunk sway, and have higher scores for chorea, bradykinesia and aggression, as well as lower cognitive scores (Grimbergen et al., 2008). It is speculated that excessive choreatic trunk movements typical for HD patients are the main cause of increased postural sway that may exceed the limits of stability and lead to falls (Grimbergen et al., 2008). To our knowledge, no literature exists relating falls to neural substrates in this specific sub-population. The TMT test has shown efficacy as a predictor of falls in HD patients, with a sensitivity of 74% and specificity of 60% (Kloos et al., 2010). Furthermore, due to the classic musculoskeletal and neurocognitive deficits exhibited by HD patients, the BBS, FRT, PPT, as well as TUG, all appear to be suitable as fall assessment tools in this population (Grimbergen et al., 2008; Busse et al., 2009; Rao et al., 2009; Quinn et al., 2013).

Regarding cognitive ability, individuals with HD have considerable difficulties multitasking and most falls occur under such circumstances (Grimbergen et al., 2008). Also, extrinsic factors such as medication, the type of walking aid, and environmental influences can all contribute to falling in HD patients (Kalkers et al., 2021). Despite the high frequency of falls in HD patients, there is a critical lack of studies on fall prevention strategies within this group. To our knowledge, only one study showed positive effects of an intensive rehabilitation program that involved respiratory exercises and speech therapy, as well as physical and cognitive exercises in fall reduction (Zinzi et al., 2007).

## 4. Discussion

**FIGURE 1:**
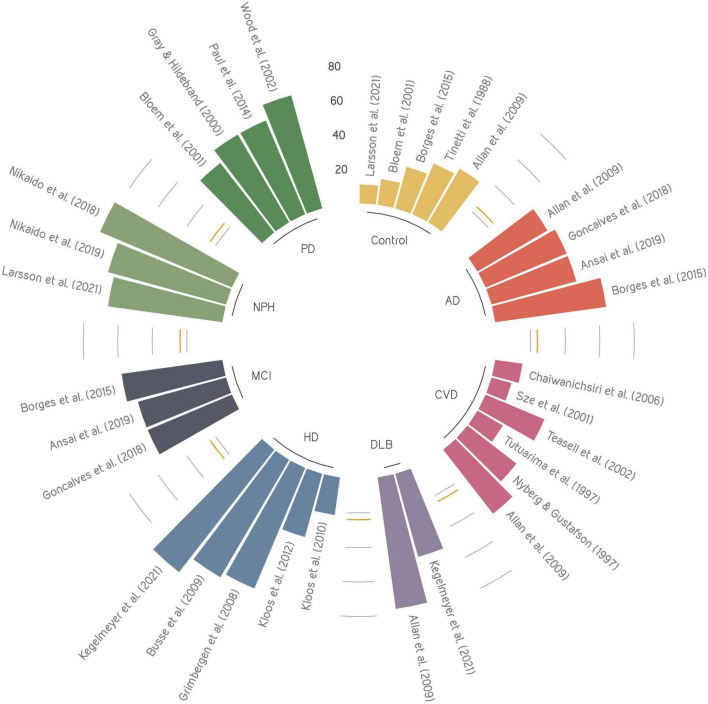
Overview of fall prevalence across cognitive disorders presented in the literature. Each color represents a cognitive disorder, where the size of each bar shows the reported fall prevalence. The yellow line represents the mean prevalence of control group. AD, Alzheimer’s disease; CVD, Cerebrovascular disease; DLB, dementia with Lewy bodies; HD, Huntington’s disease; MCI, mild cognitive impairment; NPH, normal pressure hydrocephalus; PD, Parkinson’s disease.

This comprehensive review of the literature is the first to examine the relationships between falling and various cognitive disorders. Specifically, we have compared fall prevalence (Figure 1), fall risk factors (Table 1), fall risk assessments (Table 2), and fall prevention strategies (Table 3) across different cognitive profiles. Providing the state-of-knowledge regarding efficacy of fall risk assessment tools and fall prevention strategies will support clinical decision-making toward reducing the number of falls and related injuries.

**TABLE 1:** Risk factors for falls specific to different cognitive disorders.

MCI	AD	CVD	DLB	PD	NPH	HD
Poor dual task performance (Ansai et al., 2019)	Visuospatial deficits (Ansai et al., 2019; Oki et al., 2021)	Heart disease (Tutuarima et al., 1997)	Multiple falls (Ballard et al., 1999)	Orthostatic hypotension (Gray and Hildebrand, 2000; Scharre et al., 2016)	Depression (Larsson et al., 2021)	Bradykinesia (Grimbergen et al., 2008)
Depression (Ansai et al., 2019)	Reduced balance (Oki et al., 2021)	Cognitive decline (Tutuarima et al., 1997)	Visuospatial deficits (Scharre et al., 2016)	Freezing of gait (Gray and Hildebrand, 2000; Paul et al., 2014; Fasano et al., 2017)	Cognitive decline (Larsson et al., 2021)	Chorea (Grimbergen et al., 2008)
Fear of falling (Uemura et al., 2014; Borges Sde et al., 2015)	Neuroleptic drug use (Horikawa et al., 2005)	Urinary incontinence (Tutuarima et al., 1997)	Hallucination (Scharre et al., 2016)	Dyskinesia (Paul et al., 2014)	Temporal gait variability (Nikaido et al., 2019)	Aggressiveness (Grimbergen et al., 2008)
Self-criticism of impaired cognition (Kalbe et al., 2005)		Hemineglect (Tsur and Segal, 2010)	Sleepiness (Scharre et al., 2016)	Motor fluctuations (Fasano et al., 2017)	Worse dynamic balance function (Nikaido et al., 2019)	Cognitive decline (Grimbergen et al., 2008)
		Visuospatial deficits (Tsur and Segal, 2010)	Orthostatic hypotension (Scharre et al., 2016)	Trunk flexion (Fasano et al., 2017)		Decreased gait velocity (Grimbergen et al., 2008)
		Paralysis (Tsur and Segal, 2010)	Executive deficits (Scharre et al., 2016)	Loss of arm swing (Wood et al., 2002)		Stride length (Grimbergen et al., 2008)
		Hypoesthesia (Tsur and Segal, 2010)		Reduced leg muscle strength (Paul et al., 2014)		Increased trunk sway (Grimbergen et al., 2008)
		Impulsivity (Rapport et al., 1993)		Cognitive decline (Paul et al., 2014)		
		Postural sway (Sackley, 1991)				

AD, Alzheimer’s disease; CVD, cerebrovascular disease; DLB, dementia with Lewy bodies; HD, Huntington’s disease; MCI, mild cognitive impairment; NPH, normal pressure hydrocephalus; PD, Parkinson’s disease.

**TABLE 2:** Fall risk assessments shown to be effective in cognitive disorders.

MCI	AD	CVD	DLB	PD	NPH	HD
TUG [80%, 61%] (Goncalves et al., 2018)	—	ReFR [71%, 58%] (Guimaraes et al., 2020)	FRI-21[N/A] (Tsujimoto et al., 2021)	TMT [76%, 66%] (Kegelmeyer et al., 2007), [71%, 79%] (Contreras and Grandas, 2012)	FRQ [96%, 48%] (Davis et al., 2021)	TUG [N/A] (Quinn et al., 2013)
						TMT [74%, 60%] (Kloos et al., 2010)
PPT [N/A] (Sungkarat et al., 2017)		SAFR [78%, 63%] (Breisinger et al., 2014), [48%, 77%] (Yang et al., 2021)		BBS [64%, 67%] (Schlenstedt et al., 2016)		BBS [N/A] (Grimbergen et al., 2008; Quinn et al., 2013)
		Fall Harm Risk Screen [57%, 48%] (Breisinger et al., 2014)		Hoehn and Yahr staging [77%, 71%] (Contreras and Grandas, 2012)		PPT [N/A] (Quinn et al., 2013)
		Morse Fall Scale [46%, 68%] (Yang et al., 2021)		FAB [67%, 58%] (Schlenstedt et al., 2016)		

AD, Alzheimer’s disease; BBS, Berg Balance Scale; DLB, dementia with Lewy bodies; FAB, fullerton advanced balance; FRI-21, Fall Risk Index-21; FRQ, Fall Risk Questionnaire; HD, Huntington’s disease; MCI, mild cognitive impairment; NPH, normal pressure hydrocephalus; PD, Parkinson’s disease; PPT, physical performance test; ReFR, recurrent fall risk scale; TMT, Tinetti Mobility Test; TUG, timed up and go. Sensitivities and specificities of fall risk assessments are reported in brackets: [sensitivity, specificity].

**TABLE 3:** Potential fall prevention strategies conducted in cognitive disorders.

MCI	AD	CVD	DLB	PD	NPH	HD
Tai Chi training (Sungkarat et al., 2017)	Salsa dancing (Abreu and Hartley, 2013)	Dual-task program (Zhou et al., 2021; Spano et al., 2022)	—	Argentine tango (Peter et al., 2020)	Ventriculo-peritoneal shunting (Mantovani et al., 2021)	Rehabilitation program incl. respiratory exercises, speech therapy, physical and cognitive exercises (Zinzi et al., 2007)
				Dual-task program (Silva et al., 2021)		
Exercise program involving aerobic, resistance and balance (Thaiyanto et al., 2021)	Exercise program involving balance, strengthening and walking (Suttanon et al., 2013)	VR training (Park et al., 2017)		Rehabilitation combining movement strategy and strength (Morris et al., 2015)		
Multifactorial, cognitively-based program (Fischer, 2021)	Training program combining motor and cognitive exercises (Hernandez et al., 2010)			Qigong (Song et al., 2017)		
Sensor-based balance training (Schwenk et al., 2016)	Physical exercise program (Pitkala et al., 2013)			Tai Chi (Gao et al., 2014; Song et al., 2017)		
Donepezil treatment (Montero-Odasso et al., 2019)				Balance training (Smania et al., 2010)		
				Pharmacological treatment (Giladi, 2008)		

AD, Alzheimer’s disease; CVD, cerebrovascular disease; DLB, dementia with Lewy bodies; HD, Huntington’s disease; MCI, mild cognitive impairment; NPH, normal pressure hydrocephalus; PD, Parkinson’s disease. Out of the 22 reported studies, only four presented effect sizes on fall prevention strategies (Zhou et al., 2021: WMD = 1.9; Park et al., 2017: *r* = 0.8; Gao et al., 2014: *G* = −0.4; Smania et al., 2010: Cohen’s d = −0.02).

Falls are highly prevalent among the elderly population, particularly those who are cognitively impaired. Patients with cognitive impairment may experience between 2 and 20 times more falls than asymptomatic older individuals. For example, while MCI patients fall nearly twice as often as healthy individuals (Tinetti et al., 1988; Borges Sde et al., 2015; Ansai et al., 2019), it is estimated that patients with iNPH or PDD experience up to fifteen (Larsson et al., 2021) or even twenty (Allan et al., 2009) times more falls, respectively. It is thought that the increased falls risk in iNPH and PDD patients may be related to disease-specific deficits that are different from the general population and other cognitive disorders [e.g., apraxia of gait in NPH (Adams et al., 1965) or freezing of gait in PDD (Gray and Hildebrand, 2000; Fasano et al., 2017)]. However, the fact that PDD patients fall nearly twice as often as PD patients without dementia (Gray and Hildebrand, 2000; Bloem et al., 2001; Wood et al., 2002; Allan et al., 2009) confirms that fall rates are also closely related to cognitive dysfunction.

Although fall risk factors have been well investigated throughout healthy aging, it has become clear from this review that fall-related parameters differ between cognitive disorders and thus should be evaluated in light of the individual’s specific pathology. For instance, falls in MCI patients are associated with poor dual-task performance (Ansai et al., 2019), depression (Ansai et al., 2019), fear of falling (Borges Sde et al., 2015), and self-criticism (Kalbe et al., 2005), whereas falls in AD patients are related to anosognosia (Kalbe et al., 2005) and visuospatial deficits (Ansai et al., 2019; Oki et al., 2021). Interestingly, fall risk factors in MCI and AD patients tend to be specific to the cognitive impairment, rather than their motor deficits. In contrast, for other diseases such as iNPH, PD, and HD, the majority of fall risk factors seem to be associated with impaired gait and balance. For instance, reduced lower limb muscle strength, pathological forward trunk flexion, freezing of gait, and dyskinesia are specific fall risk factors in PD, temporal gait variability and dynamic balance function are risk factors for falls specific to iNPH (Fasano et al., 2017; Nikaido et al., 2019), while excessive choreatic trunk movement plays a substantial role in the pathophysiology of falls in HD (Grimbergen et al., 2008). However, it is still not fully understood why for certain pathological conditions cognitive deficits are primary risk factors for falls, while for others, diminished physical performance is the key indicator. Some scientists present cognitive and gait impairments as two often coexisting but independent risk factors for falls (Amboni et al., 2013), while others identify these two conditions as closely related to each other (Verghese et al., 2007). It is entirely plausible, however, that the neurological site of degeneration is key to understanding these differences. In this review, we have highlighted that variability in the neural correlates of cognitive disorders manifests itself in different levels of risk and actual incidence of falling. For example, cognitive deficits such as memory impairment associated with increased fall risk in MCI and AD patients appears to be mediated by reduced volume of various brain regions such as the hippocampus (Allali et al., 2016, 2020; Keleman et al., 2020; Huang et al., 2022). Indeed, a series of studies have shown that MCI patients who have smaller hippocampal volume perform worse on the TUG test (Allali et al., 2016), while fallers with AD also have smaller hippocampal volumes compared with AD non-fallers (Keleman et al., 2020). In addition to hippocampal atrophy, the association of memory deficits with increased fall risk among people with MCI and AD might also be explained by additional neuro-mechanisms such as the accumulation of β-amyloid and neurofibrillary tangles in the brain. Indeed, higher levels of Pittsburgh compound B retention (reflecting amyloid depositions in the brain) and CSF levels of t-tau (reflecting neuronal damage) are associated with an earlier time to first fall (Stark et al., 2013). In regards to movement disorders such as PD and HD, reduced locomotor abilities associated with increased fall risk might be mediated by other neuro-mechanisms such as loss of cholinergic projections (Sarter et al., 2014). Specifically, PD fallers have significantly decreased thalamic cholinergic innervation compared with PD non-fallers (Bohnen et al., 2009). The interplay between cognition, gait, and falls may also reflect damage in shared brain regions for both cognitive and motor disorders. For example, white matter lesions are associated with reduced cognitive performance, impaired mobility and falls (Bolandzadeh et al., 2012), and are present in people with MCI (Snir et al., 2019), dementia (Taylor et al., 2019), PD (Lee et al., 2010), and HD (Casella et al., 2020). The more pronounced presence of white matter hyperintensities in PDD patients compared with PD patients without dementia, suggests that white matter lesions may be a contributing factor for cognitive decline (Lee et al., 2010). Accordingly, researchers should consider the neuro-mechanisms associated with both gait deficits and the cognitive status when evaluating risk factors for falls and improving fall risk assessment tools and fall prevention strategies.

Reliable fall risk assessment tools that are specific to each condition may enable the identification of fallers at an earlier time point and help target effective fall prevention strategies. Although different risk assessment tests exist, our review suggests that there is no unified tool that considers all contexts. For instance, the TUG test is more sensitive for assessing DLB fallers than those with HD (Kegelmeyer et al., 2021), and can be used as a predictor of falls in MCI but not in AD (Goncalves et al., 2018) or NPH (Davis et al., 2021). Similarly, PPT may be used for assessing fall risk of patients with HD (Quinn et al., 2013) but not AD (Farrell et al., 2011; Ryan et al., 2011). As such, different fall risk assessment tools seem to detect aspects that are functionally meaningful to some specific cognitive disorders but not others, possibly because they focus on gait abnormalities rather than cognitive function.

Fall prevention strategies that have been successful in cognitively normal adults have failed in patients with cognitive impairments, suggesting that such strategies should be carefully prescribed to the target population (Shaw, 2007). In this respect, emphasizing a therapeutic approach addressing fear of falling as well as dual tasking (Ansai et al., 2019; Thaiyanto et al., 2021) in MCI patients could be a beneficial component for strategies to prevent falls in this group, but such approaches may not be successful in other diseases such as AD. Indeed, AD patients show a much lower prevalence of fear of falling (Borges Sde et al., 2015) and no differences in dual-task performance have been found between fallers and non-fallers (Goncalves et al., 2018; Ansai et al., 2019). Furthermore, different dance styles have been recommended as fall prevention programs not only in healthy (Jeon et al., 2005; Alpert et al., 2009; Eyigor et al., 2009; Noopud et al., 2019), but also in pathological (Abreu and Hartley, 2013; Peter et al., 2020) aging. Tai Chi training is approximately twice as effective in reducing the risk for falls in both MCI and PD patients compared with healthy individuals (Gao et al., 2014; Sungkarat et al., 2017), but has not been investigated in other cognitive disorders. For AD (Ansai et al., 2019; Oki et al., 2021), DLB (Scharre et al., 2016) and CVD (Nyberg and Gustafson, 1997; Tsur and Segal, 2010) patients, interventions that aimed to enhance visuospatial abilities are believed to ameliorate treatment. Although gamification of training has received increasing attention in supporting healthy aging and fall prevention (Verghese et al., 2010; Smith-Ray et al., 2015), its effectiveness has been reported only in post-stroke patients (Park et al., 2017). This review demonstrates that the efficacy of fall prevention strategies clearly differs between cognitive disorders. However, to date, fall prevention strategies have rarely shown to be effective in reducing fall rates. As such, and based on the evidence presented in this review, we strongly recommend that cognitive training is included in fall prevention strategies to enhance their efficacy, especially in patients with dementia.

In view of increased health complications and the considerable costs of care for patients with cognitive impairment who have fallen (Florence et al., 2018; Haddad et al., 2019), there is a critical unmet need to optimize fall risk assessments and prevention strategies, particularly for this population. In this review, we show that fall-related characteristics differ between cognitive disorders, and we therefore strongly recommend that interventions for each patient group should be specifically targeted and optimized. Moreover, there is an urgent need to further study the specific predictors of falls across various types of cognitive disorders to enable more tailored and effective fall risk assessment tools to be developed. Such combined approaches may not only improve the quantification of fall risk early in the disease and target effective fall prevention, but also help to reduce morbidity and decrease the socioeconomic burden associated with falls.

## Author contributions

KM reviewed the literature and wrote the first draft of the manuscript. GC, WT, and VS edited and contributed to the final manuscript. All authors contributed to the article and approved the submitted version.
